# Higher biodiversity is required to sustain multiple ecosystem processes across temperature regimes

**DOI:** 10.1111/gcb.12688

**Published:** 2014-08-18

**Authors:** Daniel M Perkins, R A Bailey, Matteo Dossena, Lars Gamfeldt, Julia Reiss, Mark Trimmer, Guy Woodward

**Affiliations:** 1 Department of Life Sciences, Imperial College LondonSilwood Park Campus, Berkshire, SL5 7PY, UK; 2 School of Mathematical Sciences, Queen Mary University of LondonLondon, E1 4NS, UK; 3 School of Mathematics and Statistics, University of St AndrewsSt Andrews, Fife, KY16 9SS, UK; 4 School of Biological and Chemical Sciences, Queen Mary University of LondonLondon, E1 4NS, UK; 5 Department of Biological and Environmental Sciences, University of GothenburgBOX 461, Gothenburg, SE-40530, Sweden; 6 Department of Life Sciences, Whitelands College, University of RoehamptonLondon, SW15 4JD, UK

**Keywords:** ecosystem functioning, environmental warming, functional redundancy, multifunctionality, species richness

## Abstract

Biodiversity loss is occurring rapidly worldwide, yet it is uncertain whether few or many species are required to sustain ecosystem functioning in the face of environmental change. The importance of biodiversity might be enhanced when multiple ecosystem processes (termed multifunctionality) and environmental contexts are considered, yet no studies have quantified this explicitly to date. We measured five key processes and their combined multifunctionality at three temperatures (5, 10 and 15 °C) in freshwater aquaria containing different animal assemblages (1–4 benthic macroinvertebrate species). For single processes, biodiversity effects were weak and were best predicted by additive-based models, i.e. polyculture performances represented the sum of their monoculture parts. There were, however, significant effects of biodiversity on multifunctionality at the low and the high (but not the intermediate) temperature. Variation in the contribution of species to processes across temperatures meant that greater biodiversity was required to sustain multifunctionality across different temperatures than was the case for single processes. This suggests that previous studies might have underestimated the importance of biodiversity in sustaining ecosystem functioning in a changing environment.

## Introduction

Biodiversity loss and environmental warming are major threats to the functioning of natural ecosystems (MEA, 2005; IPCC 2013), with both having potentially strong impacts on key components of ecosystem functioning, such as decomposition or primary production (Hooper *et al*., 2012). However, surprisingly little is known about the combined effects of biodiversity loss and temperature on single and joint ecosystem processes, given that each process may respond differently to these drivers of change (Duffy, 2009; Yvon-Durocher *et al*., 2010; Hooper *et al*., 2012). Furthermore, as both species loss and warming are widely predicted to increase simultaneously in many ecosystems worldwide in the near future, understanding the interactions between them will be critical for predicting the future levels of ecosystem functioning (Cardinale *et al*., 2012).

Intensive research into biodiversity–ecosystem functioning (hereafter B–EF) relationships over the past two decades has found that in general a few species are required to maximize single ecosystem processes (Cardinale *et al*., 2006, 2012). For instance, in freshwater communities, B–EF curves can saturate at just six species (Jonsson & Malmqvist, 2003) or fewer (Perkins *et al*., 2010; Reiss *et al*., 2010, 2011), a tiny fraction of the real biodiversity found in natural systems. Such studies suggest that many species are functionally redundant, although this has been questioned recently, in part because of the lack of studies that consider multiple processes, which provide a more complete picture of ecosystem functioning (Gamfeldt *et al*., 2008; Reiss *et al*., 2009). A few recent studies suggest that high levels of biodiversity may be needed to sustain multifunctionality (Duffy *et al*., 2003; Hector & Bagchi, 2007; Gamfeldt *et al*., 2008; Zavaleta *et al*., 2010; Isbell *et al*., 2011; Peter *et al*., 2011; Maestre *et al*., 2012a,b). Positive biodiversity–ecosystem multifunctionality (hereafter B–MF) relationships can arise through variation among species in their contributions to different processes, and/or through interactions among species that enhance multiple processes (Gamfeldt *et al*., 2008). While this area of research is starting to gain momentum, the relative importance of both these effects remains unclear (Byrnes *et al*., 2014). A better mechanistic understanding can be gained by coupling controlled experiments with appropriate analyses of both multifunctionality and its component processes (Reiss *et al*., 2009; Byrnes *et al*., 2014).

The importance of biodiversity for multifunctionality should be especially critical in a heterogeneous or changing environment. This is because species differ in their optima (where physiological processes are maximized) and therefore their contribution to ecosystem functioning across environmental gradients (Isbell *et al*., 2011; Steudel *et al*., 2012). Despite ecosystem processes being strongly temperature-dependent (Brown et al., 2004), and the widespread concern about the effects of environmental warming (IPCC 2013), no study (of which we are aware) has tested the effects of temperature on B–MF relationships. These are likely to be particularly pronounced in aquatic systems because they are dominated by ectotherms, whose performances are largely determined by environmental temperature (Vannote & Sweeney, 1980). In these systems, we might expect significant variation in the identity of species, or species assemblages, contributing to processes under different thermal regimes (Woodward *et al*., 2010).

We used a model freshwater system to test the impacts of temperature and biodiversity on both multiple individual processes and ecosystem multifunctionality. Our experiments were conducted at three temperatures (5, 10 and 15 °C) selected to match the annual average and seasonal extremes of streams within the temperate study region and to include 5 °C increments that mimic the extent of warming predicted by 2100 (IPCC 2013), i.e. from 5 °C to 10 °C and from 10 °C to 15 °C. At each temperature, we manipulated the richness of four dominant benthic macroinvertebrate species varying in feeding preferences (see Materials and methods). We quantified rates of five key ecosystem processes, ranging from resource depletion (leaf decomposition and algal consumption [hereafter herbivory]) to production of fine particulate organic material (hereafter FPOM), and algae biomass, and the regeneration of the macronutrient nitrogen (N) through ammonification (Ammonium [NH_4_^+^]).

Our objectives were first to test the dual effects of species richness and temperature on single ecosystem processes and then to quantify their combined contribution to multifunctionality. To do so, we included a range of novel ‘Type’ models in the analysis of single processes, which we have recently developed for B–EF research (Reiss *et al*., 2011). These models are based on the general assumption that a species performance in polyculture can be predicted from its performance in monoculture and that temperature shapes the performance of a species in polyculture in the same way as it does in monoculture (Table1).

**Table 1:** Array of linear models used to test the effects of species diversity and environmental temperature on single ecosystem processes

anova term	Number of parameters	Explanation if significant (*P* < 0.05)	d.f
a) Constant	**1**	The grand mean is different from zero.	1
b) Temperature	**3**: 5, 10 and 15 °C	Environmental temperature influences functioning (one or more levels differ from grand mean).	2
c) Richness	**4**: 1, 2, 3 and 4 species cultures	Species number influences functioning (one or more levels differ from grand mean).	3
d) Type	**4**:*y = a*_*1*_*x*_*1*_* + a*_*2*_*x*_*2*_ *+ a*_*3*_*x*_*3*_ *+ a*_*4*_*x*_*4*_	Polyculture (*y*) performance is well predicted from monoculture information.	3
e) Composition	**15**: Assemblages: A, B, C, D, AB, AC, etc.	Species assemblages perform differently (variation above that accounted for by terms c & d).	8
f) Richness × Temperature	**12**: (4 × 3)	Different species richness effects emerge at different temperatures (variation above that accounted for by terms b & c).	6
g) Type × Temperature	**12**: 5 °C: *y = b*_1_*x*_1_ + *b*_2_*x*_2_ + *b*_3_*x*_3_ *+ b*_4_*x*_4_	Species perform in an additive fashion, but performance changes with temperature (variation above that accounted for by terms b & d).	6
	10 °C: *y = c*_1_*x*_1_ + *c*_2_*x*_2_ + *c*_3_*x*_3_ *+ c*_4_*x*_4_		
	15 °C: *y = d*_1_*x*_1_ + *d*_2_*x*_2_ + *d*_3_*x*_3_ *+ d*_4_*x*_4_		
h) Composition × Temperature	**45**: (15 × 3)	The effects of composition varies with temperature (variation above that accounted for by terms e, f and g).	16

anova terms are listed in increasing complexity (number of parameters), starting with the smallest (‘Constant’), up to the largest (‘Composition × Temperature’). Each letter (a–h) corresponds to the edge (connection) between models in the hierarchy of models (see Figure S3 for how models are related). Our statistical analysis was designed in a way that the explanation given by the significance of terms in the anova table reflects the comparison between the sums of squares for that term and the sum of squares for its (simpler) constituent parts, which is reflected in the degrees of freedom (d.f) for that term. Constants such as *a*_1_ are the fitted parameters for species 1–4 and *x*_*i*_ is the number of individuals of type *i* in the culture (for example, in the duoculture AB, *x*_1_ =* x*_2_ = 6 and *x*_3_ =* x*_4_ = 0).

We also extended the recent ‘Multiple Threshold’ framework of Byrnes *et al*. (2014) to the analysis of multifunctionality at the different experimental temperatures. This framework describes the linear relationship between species richness and the total number of processes exceeding a predetermined threshold (some proportion of maximal functioning). In contrast with other approaches introduced to investigate B–MF relationships (Hooper & Vitousek, 1998; Hector & Bagchi, 2007; Gamfeldt *et al*., 2008), the one used here investigates the effect of diversity on multifunctionality across a range of thresholds and circumvents the problem of arbitrary thresholds being defined by the investigators (e.g. Zavaleta *et al*., 2010; Maestre *et al*., 2012b).

The combination of our experimental design, novel statistical models, and the model framework we adopted allowed the actual species level contribution to specific processes and multifunctionality to be tested. This improves on previous studies that have calculated (Gamfeldt *et al*., 2008) or estimated individual species contributions to multifunctionality using regression-based techniques (Hector & Bagchi, 2007; Isbell *et al*., 2011). Consequently, we were able to characterize the links between single processes and multifunctionality and their responses to biodiversity and temperature, and to test the following predictions.

For single ecosystem processes, we predicted that: (i) species effects should be additive with polyculture performance well approximated by the sum of monoculture parts (Reiss *et al*., 2011); (ii) process rates should increase with biomass (Brown et al., 2004) if all species contribute to a given process; and (iii) species contribution to processes should vary with temperature and differ among species (Vannote & Sweeney, 1980), and thus models including temperature should predict process rates more accurately.

For multiple processes, we predicted that: (i) multifunctionality should increase with species richness, with a different species pool driving processes at different temperatures because species possess different functional and response traits (Vannote & Sweeney, 1980; Petchey & Gaston, 2002); and (ii) B–MF relationships are sensitive to the choice of threshold values, so the strength of biodiversity effects should vary across a range of multifunctionality thresholds (Byrnes *et al*., 2014).

## Materials and methods

### Experimental set-up

Laboratory experiments were conducted in aquaria (28 × 14 × 20 cm, volume 5 l) in environmental-control (EC) rooms maintained at 5, 10 or 15 °C (±1 °C). Aquaria were filled with 1 : 3 parts circumneutral stream/degassed and dechlorinated tap water (Perkins *et al*., 2010; Reiss *et al*., 2011), aerated, and arranged in a block design under full-spectrum lighting (∽50 μmol photons m^−2^ s^−1^). Photoperiod was set to resemble late autumn conditions (8 h light/16 h dark cycle) when the experiment took place. Logistical constraints meant that we designed the experiment to explicitly quantify the interactions between temperature and biotic drivers (the number, type and composition of species), rather than investigate the effects of temperature *per se* (i.e. the relative effect of temperature on process rates). To provide a valid statistical test for the latter would have required unattainable levels of replication of EC rooms. Within each temperature regime, we manipulated the richness of four benthic macroinvertebrate species that are widespread and codominant members of local stream assemblages; *Asellus aquaticus* (L.)*, Bithynia tentaculata* (L.), *Gammarus pulex* (L.) and *Sericostoma personatum* (Kirby & Spence). These consumer species represent a range of feeding preferences from obligate detritivores [*S. personatum* (Elliott, 1969)], facultative detritivore-herbivores [*A. aquaticus* and *G. pulex* (Moore, 1975; Graça *et al*., 1993)] to obligate herbivores [*B. tentaculata* (Brendelberger, 1995)] that exploit the ‘brown’ (i.e. detrital) and/or ‘green’ (i.e. algal) energy pathways in the food web (Woodward *et al*., 2008).

Consumer diversity (all monocultures, and all possible equal combinations of two, three and four species assemblages) was manipulated in a substitutive design with a constant density of 12 individuals per aquarium (Jonsson & Malmqvist, 2000; Perkins *et al*., 2010; Reiss *et al*., 2011). We also included a microbe-only control treatment to test if process rates in these treatments differed to those when macroinvertebrate consumers were present (Data S1). These diversity treatments were crossed with temperature to give 48 experimental treatments and replicated to give a total of 96 aquaria. Although we had only two replicates for each experimental treatment, replication for each level of richness and the number of treatments containing the same species were high, as is typical for such factorial diversity experiments (Bailey & Reiss, 2014). For example, in our experiment, each species was present in half (48/96) of the experimental units. Assemblage biomass was calculated for each aquarium from high-resolution digital photographs taken of each individual consumer, measured using image analysis software Image-Pro® Plus (Media Cybernetics, Inc., Rockville, MD, USA) and converted into dry body mass (mg) using empirically derived length–mass equations (see Data S1 for equations).

Each aquarium was supplied with two basal resources: 3 g of freshly abscised air-dried alder leaves [*Alnus glutinosa* L. Gaertn] preconditioned in invertebrate-free aquaria for 7 days previously (cf. Perkins *et al*., 2010; Reiss *et al*., 2011) and a 10 × 10 cm ceramic tile colonized by benthic algae (*Navicula cryptonella* Lange-Bertalot). *Navicula cryptonella* was cultured on tiles for 3 weeks prior to the experiment in sterile tanks containing nutrient-rich diatom culture medium (CCAP; http://www.ccap.ac.uk/media/documents/DM.pdf) until a dense monospecific biofilm was achieved (mean chlorophyll concentration 3.70 μg cm^−2^, ± 0.15 SE). Both these food sources represent widespread basal resources for many freshwater food webs, including those in the surrounding locale, which support diverse assemblages of detritivore and herbivore consumers (e.g. Woodward *et al*., 2008).

The experiment ran for 32 days, by which time depletion of resources in the fastest treatments approached 50% of initial standing stocks (cf. Perkins *et al*., 2010; Reiss *et al*., 2010, 2011). Five ecosystem processes were measured over the course of the experiment: leaf decomposition, herbivory, algal production, FPOM production and ammonification (NH_4_^+^). Rates of these processes were calculated from the change in stocks or concentrations from time zero (*T*_*0*_), when invertebrate assemblages were added, to the end of the experiment, except for ammonification, which was calculated between *T*_*0*_ and *T*_*8*_ (see below). Leaf decomposition was quantified from the material remaining (>1 mm diameter) at the end of the experiment, which was dried at 80 °C to a constant weight and subtracted from initial values – after accounting for losses caused by leaching and microbial activity prior to the addition of invertebrate assemblages (see Data S1). Algal biomass remaining on the tiles at the end of the experiment was scraped into individual bottles and chlorophyll analysis was performed (Lorenzen, 1967). To measure rates of herbivory, these chlorophyll concentrations were subtracted from initial concentrations (quantified for 30 additional tiles) at the beginning of the experiment. FPOM production was quantified from organic material <1 mm diameter, collected from each aquarium, dried and weighed. To quantify algal production, we placed a blank 8 × 8 cm ceramic tile on the bottom of each aquarium, which was enclosed in a fine mesh cage (0.25 mm aperture) to prevent consumer grazing. Algal biomass was removed from these tiles at the end of the experiment and chlorophyll analysis performed (as described above). Ammonification was quantified between *T*_*0*_ and *T*_*8*_ when NH_4_^+^ peaked in the water column (Figure S1). NH_4_^+^ concentrations were determined in 15 ml water samples filtered through a preflushed (20 ml ultra-high-purity water, Elga) polypropylene membrane filter (0.2 μm, VWR International, Leicester, UK) and analysed using a segmented flow auto analyser (Skalar, Netherlands) and standard techniques (Grasshoff *et al*., 1983).

### Statistical analysis of single processes

Single processes were analysed using a series of linear models that included terms for the effects of environmental temperature (‘Temperature’), species richness (‘Richness’), assemblage composition (‘Composition’) and their interactions. In our analysis, ‘Richness’ reflects the average contribution of species number to a process, irrespective of the particular species present, and ‘Composition’ reflects the average contribution of different species assemblages to a process (Jonsson & Malmqvist, 2000; Perkins *et al*., 2010).

We also included a set of ‘Type’ models into the analysis to test explicitly for additive species effects in the experiment (after Reiss *et al*., 2011). The simplest of these models (‘Type’) assumes that each species has a unique performance that provokes a characteristic effect on a process, irrespective of whether the species is combined with other species or not. Thus, the rate of a given process is equivalent to: *y = a*_*1*_*x*_*1*_* + a*_*2*_*x*_*2*_* + a*_*3*_*x*_*3*_* + a*_*4*_*x*_*4*_, where *a*_*i*_ is the performance of species *i* in monoculture and *x*_*i*_ is the number of organisms of species *i* in an aquarium (defined as covariates x_1_,..,x_4_). We also included the larger model ‘Type × Temperature’ which maintains the assumption of additive species effects, but the effects of species are different for each level of temperature (Table1).

In total, we considered 18 models, all of which were related in a hierarchy (as shown in Figure S2) and were fitted by analysis of variance (anova). For each process, we ranked all models in terms of parsimony by calculating Akaike's Information Criterion (AIC_c_) with correction for finite sample sizes (after Hurvich & Tsai, 1989; Table S1). Because the largest model in our analysis was defined by ‘Composition × Temperature’ yet some of the smaller models included covariates (e.g. ‘Type’), there was no statistical package to run the whole suite of models in a single pass. The standard procedure in such circumstances is to extract the output from the individual models and use the residual sums of squares (SS) and degrees of freedom (d.f.) to build the anova table (e.g. Bell *et al*., 2005; Reiss *et al*., 2011). Each row in the anova table corresponds to a specific hypothesis (given in Table1) and tests for whether the difference between a model, and its related smaller ones, can explain the data significantly better or not (Grafen & Hails, 2002; Reiss *et al*., 2011).

In all the models, we included two random error terms: one for blocks (6 levels); and one for EC rooms (3 levels), with ‘Blocks’ nested in ‘Rooms’. Because the whole of each EC room had to be at the same temperature, the ‘Temperature’ factor was effectively the whole-plot factor in a split-plot experiment (Bailey, 2008; Montgomery, 2012). As there were the same number of rooms as temperatures, there are no degrees of freedom for estimating the variability between rooms, and hence no denominator for an F-test of the null hypothesis that ‘Temperature’ had no effect. For each of the five single ecosystem processes, there was at least one interaction involving ‘Temperature’ that was statistically significant at the 5% level (Table2). By the marginality principle (Nelder, 1977; Grafen & Hails, 2002), which is similar to the hierarchy principle (Montgomery, 2012), no interaction should be included in a fitted model without its relevant main effects. Thus, it is clear that ‘Temperature’ should be included in the fitted model, even though there is no valid statistical test for the effect of ‘Temperature’.

**Table 2:** Analysis of variance testing the effects of species diversity in combination with temperature on single ecosystem processes

anova term	d.f.	Leaf decomposition		Herbivory		FPOM production		Algal production		Ammonification	
		*F*	*P*	*F*	*P*	*F*	*P*	*F*	*P*	*F*	*P*
Temperature	2	–	–	–	–	–	–	–	–	–	–
Richness	3	0.4	0.728	3.8	0.017	6.0	0.002	0.3	0.815	0.2	0.874
Type	3	37.8	<0.001	19.3	<0.001	150.7	<0.001	30.0	<0.001	7.9	< 0.001
Composition	8	0.2	0.985	1.5	0.198	2.7	0.019	0.6	0.742	2.7	0.017
Richness × Temperature	6	1.5	0.190	1.0	0.428	1.4	0.221	4.3	0.002	0.3	0.937
Type × Temperature	6	3.8	0.004	3.4	0.008	6.0	< 0.001	3.4	0.007	4.7	0.001
Composition × Temperature	16	2.3	0.017	1.4	0.191	1.4	0.169	1.7	0.092	2.6	0.007
Blocks (Rooms)	3										
Error	42										

Each row in the anova table corresponds to one of the hypotheses in Table1. In turn, this corresponds not to a model but to the difference between the model shown in the same row of Table1 and the sum of all simpler models (see Figure S3). For example, the small *P*-values observed for the row labelled ‘Type × Temperature’ indicate that, for these data, the larger model ‘Type × Temperature’ cannot be simplified to the smaller model ‘Type + Temperature’. There is no valid statistical test for the main effect of ‘Temperature’ on processes because ‘Temperature’ and ‘Rooms’ were the same in our study (one environmental-control room per temperature; see Materials and methods).

### Analysis of multifunctionality

We applied the Multiple Threshold framework of Byrnes *et al*. (2014) in the analysis of multifunctionality. This framework uses a metric for multifunctionality (*MF*_*t*_) that describes the linear relationship between species richness and total number of processes (*P*) that exceed a predetermined threshold (*t*_*i*_), defined as a given proportion of the maximum observed rate for each process in a study:

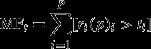
1

where *p*_*i*_ is the value for process *i* in a given unit and *r*_*i*._ is a mathematical function for standardizing processes (see below). The inverse value of MF_*t*_ estimates the proportional increase in multifunctionality per addition of a species – e.g. a MF_*t*_ value of 0.25 indicates that four additional species are needed to bring an extra process above a given threshold.

As a first stage, we defined the desirable direction of process rates and calculated the maximum rate (*R*_max_) for each process. In all cases, the best-performing aquaria were defined as those with the highest positive impact on processes and values of *R*_max_ were calculated from the average of three highest performing aquaria within each temperature level. Here, we use the mean of *n *+* *1 highest measurements of a process as our maximum, where *n* is the smallest sample size of a single richness treatment level (Byrnes *et al*., 2014). In the case of ammonification, process values were negative at 5 and 10 °C and positive at 15 °C [i.e. net uptake of NH_4_^+^ and net release of NH_4_^+^ over time, respectively (Table S2)]. The best-performing aquaria were identified as being opposite in direction to the microbe-only controls, which exhibited a net uptake of NH_4_^+^ at all temperatures (Figure S1). To standardize this process (i.e. make all process values positive), we normalized values by accounting for the range of values in the data set using the formula: (*x* – *z*)*/*(*a – z*), where *x* is the observed value, *z* and *a* are the lowest and highest observed value in the data set, respectively.

In a second stage, we used the multifunc package in the R environment (R Development Core Team, 2013) to first compute the number of processes performing at or above thresholds of 25%, 50% and 75% of *R*_max_ for each richness level within temperatures. That is, data for each temperature were analysed separately. These thresholds represent the range considered in previous studies on ecosystem multifunctionality (Gamfeldt *et al*., 2008; Zavaleta *et al*., 2010; Maestre *et al*., 2012b). We performed an F-test to assess the effects of species richness on multifunctionality at these thresholds and to test whether including species richness provided a better fit than a model with only an intercept (Byrnes *et al*., 2014).

We then fitted a generalized linear model with a quasi-poisson error to estimate a linear relationship predicting the number of processes performing at or above all thresholds (Byrnes *et al*., 2014). We restricted our analysis between thresholds of 1–83%, as above this upper threshold, the models would not converge. Slope estimates (*MF*_t_) and statistics were then computed across temperatures and plotted against threshold values. We used the *getIndices* function in multifunc package to extract specific metrics, which provide key information about how diversity can influence multifunctionality including: Minimum Threshold (*T*_min_), Maximum Threshold (*T*_max_), Threshold of Maximum Diversity Effect (*T*_mde_) and Realized Maximum Effect of Diversity (*R*_mde_) (see Fig. 2 for definitions).

To compare the performance of different assemblages across the temperature gradient, we also calculated a multifunctionality ‘index’ for each aquarium (after Zavaleta *et al*., 2010). This index is based upon the mean percentage of *R*_max_ achieved by consumer assemblages across all the processes. Within each temperature, we then ranked each assemblage composition according to this index to assess changes in performance (Table3).

**Table 3:** Multifunctionality index scores for best-performing species assemblages across temperatures including all monocultures

Assemblage composition	Multifunctionality index (rank out of 15) by temperature		
	5 °C	10 °C	15 °C
*A.a + G.p + S.p*	76% (1)	58% (9)	73% (1)
*G.p*	70% (4)	75% (1)	58% (8)
*A.a*	44% (15)	48% (13)	41% (15)
*B.t*	50% (13)	46% (14)	58% (9)
*S.p*	73% (2)	69% (3)	67% (3)

Within each temperature regime, each assemblage composition was ranked (out of 15) according to a multifunctionality index, which is the mean percentage of the *R*_max_ observed for each ecosystem process. As *R*_max_ for each process was calculated from the mean of the highest three aquaria (within each temperature level), it is possible for some assemblages to achieve >100% of this level for one or more process. Abbreviations: *A.a Asellus aquaticus*;*B.t*,*Bithynia tentaculata*;*G.p*,*Gammarus pulex* and *S.p*,*Sericostoma personatum*.

All statistical tests were performed on untransformed data from all 90 experimental aquaria (controls excluded) using R version 3.0.2 (R Development Core Team, 2013).

## Results

### Single processes

Process rates were not strongly related to species richness (Table2). While the ‘Richness’ model that tested for species richness effects was significant for rates of herbivory and FPOM production, there was no systematic pattern in functioning across richness levels (Figure S3). Overall, species richness explained very little variation in the data and the ‘Richness’ model ranked among the worst models based upon AIC_c_ (Table S1).

**Figure 1:**
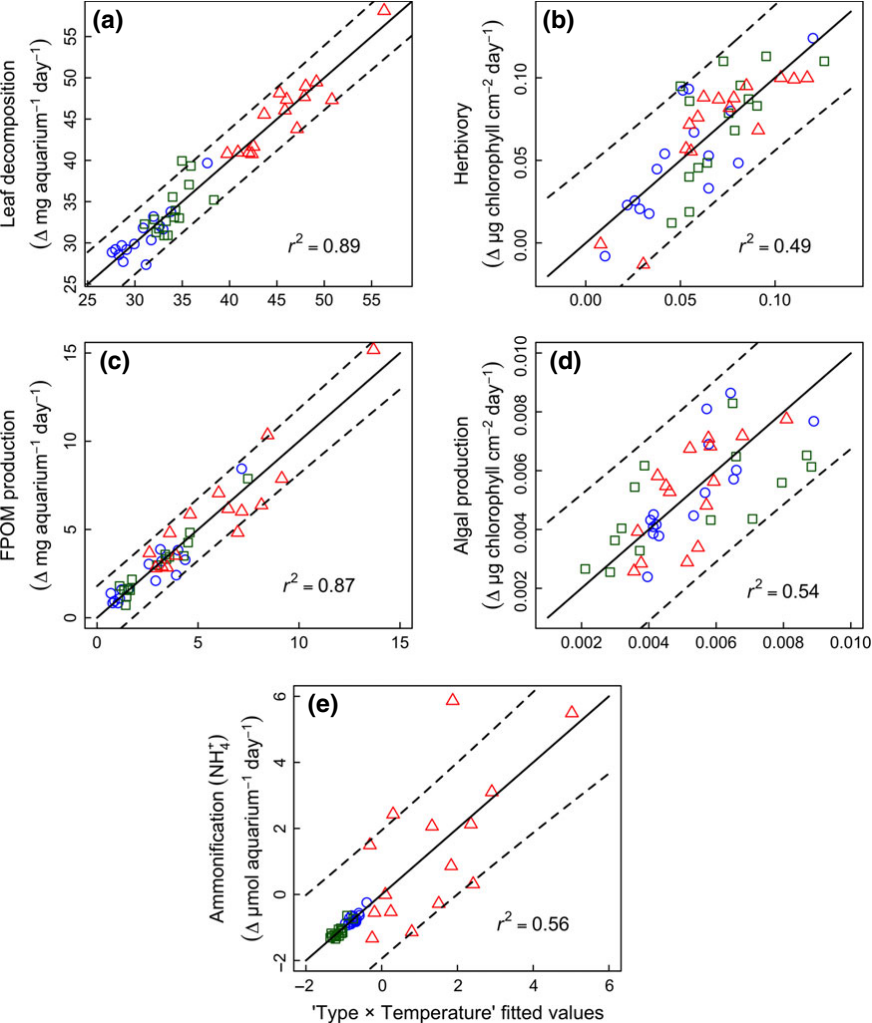
Relationships between fitted values for model ‘Type × Temperature’ and observed rates of ecosystem processes (a–e). Circles, squares and triangle symbols correspond to 5, 10 and 15 °C temperature treatments respectively. Solid lines represent 1: 1 fits and dashed lines prediction intervals (± 2 SD). Coefficient of variation values (*r*^*2*^) are given for the variation explained by the model in the analysis (Table S1).

Species effects on all single processes were largely additive and influenced by temperature, which meant that our statistical models that tested for this (‘Type’ and ‘Type × Temperature’) always needed to be included in the final model (significant for all processes; Table2). The performances of species polycultures in our experiment were well approximated by simply extrapolating from the monocultures (i.e. the polycultures were roughly ‘the sum of their parts’ with the model ‘Type’ explaining 10–54% of variation across processes; Table S1). Predictions were significantly improved, however, when information on species-specific responses to temperature were included (model ‘Type × Temperature’ explained 49–89% of variation across processes; Fig.1). For each process, ‘Type × Temperature’ ranked among the top three models and the difference between its AIC_c_ value and that for the top-ranking model was never more than 5% of the difference between the largest and smallest value (Table S1).

The superiority of the ‘Type × Temperature’ model in explaining single processes highlights that nonadditive interactions were weak in our experiment. Indeed, models ‘Composition’ and ‘Composition × Temperature’ which tested for this had only limited effects across processes (Table2) and overall were ranked among the worst models (Table S1). While we could not provide a valid statistical test for the effects of ‘Temperature’, it was clear temperature effects were positive for leaf decomposition and FPOM production, inconsistent for herbivory and net ammonification and absent for algal production (Figure S4).

Not all process rates were significantly related to assemblage biomass, highlighting that not all species contributed to each individual process. Leaf decomposition and FPOM production were maximized by monocultures of *S. personatum*, the largest species in this study (Table S2), and were positively correlated with assemblage biomass (Ordinary Least Squares regression; *r*^2^ = 0.35, *n *=* *90, *P *<* *0.001, and *r*^2^ = 0.50, *n *=* *90, *P *<* *0.001, respectively; Figure S5). In contrast, algal production was maximized in monoculture by *G. pulex* (Table S2), the smallest species in the study, and significant negative effects of assemblage biomass were observed (*r*^2^ = 0.06, *n *=* *90, *P *=* *0.011). There was no effect of assemblage biomass for herbivory and net ammonification (Figure S5).

### Multifunctionality

**Figure 2:**
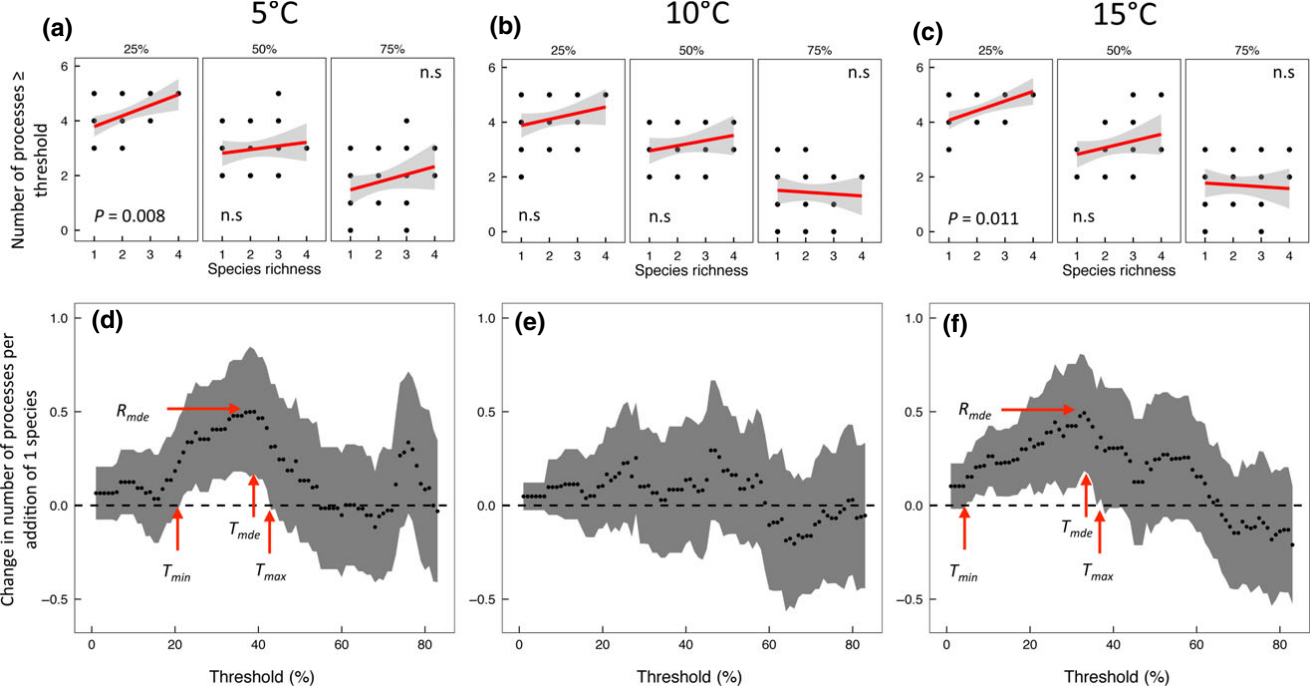
Relationships between species richness and multifunctionality at different environmental temperatures. Panels a–c show relationships for multifunctionality thresholds of 25%, 50% and 75% of maximum observed process rates (*R*_max_) with temperature. Panels d–f show the slope of the relationship between species richness and multifunctionality at multiple threshold values (1–83% of *R*_max_) for different temperatures. The 95% confidence intervals (indicated in grey) around the estimated slopes (filled data points) indicate whether the intervals contain zero, giving a test of the threshold values at which diversity has no effect on multifunctionality. *T*_min_ and *T*_max_ are the slopes with the lowest and highest threshold that is different from zero, respectively. *T*_mde_ is the threshold with the steepest slope and *R*_mde_ shows the maximum slope estimated at *T*_mde_.

Species richness was positively correlated with the number of processes exceeding threshold values of 25% at 5 °C (*F*_1,28_ = 8.04, *P *=* *0.008) and 15 °C (*F*_1,28_ = 7.49, *P *=* *0.011), but not for 50% and 75% at either temperature (*P *>* *0.05 in both cases; Fig.2a, c). In contrast, no significant relationship was observed for any of the three thresholds at 10 °C (all *P *>* *0.05; Fig.2b), highlighting that species richness effects were not ubiquitous, but dependent on the environmental context.

Examining the slope of the richness–*MF*_*t*_ relationship across our full range of thresholds revealed that richness had a positive impact at both temperature extremes (Fig.2d, f), but no effect at 10 °C (Fig.2e). At 5 °C, multifunctionality increased with species richness at thresholds between 21% (*T*_min_) and 43% (*T*_max_) and the threshold of maximum diversity effects (*T*_mde_) was 39%, with a realized maximum diversity effect (*R*_mde_) of 0.50; i.e. approximately two species were needed to drive an additional process. For 15 °C, the relationship peaked at a similar threshold (*T*_mde_ = 33%) and displayed a similar *R*_mde_ value (0.49 processes added per species) to that observed at 5 °C, yet multifunctionality increased with species richness across a greater range of thresholds (between 4% and 38%).

The identity of assemblages promoting processes changed with temperature and no single assemblage was the best at performing across all temperatures, as revealed by our multifunctionality index (Table3). For example, the assemblage of *A. aquaticus + G. pulex + S. personatum* performed best at 5 °C and 15 °C, but was only ranked 9 (of a possible 15) at 10 °C (Table3). This meant that polyculture performance decreased relative to monoculture performance at 10 °C compared to the other temperatures, and thus no significant positive richness effects were observed at this temperature (Fig.2e).

## Discussion

We found clear and compelling evidence that biodiversity becomes more important in sustaining ecosystem functioning when multiple processes and environmental contexts are considered, with species contributing differently to each process, and in ways that change with environmental conditions. A general picture emerged from our study: single processes depended largely on the additive contribution of species across temperatures, whereas multifunctionality was primarily driven by species complementarity across processes and temperatures. Our results clearly demonstrate the context dependency of biodiversity effects as, although species richness had negligible effects on individual processes, it influenced multifunctionality, but only at the coldest and the warmest temperature. This key finding highlights the need to measure multifunctionality and to do so across a range of environmental conditions, to bring greater realism and predictive power to future B–EF research (Gamfeldt *et al*., 2008; Reiss *et al*., 2009; Cardinale *et al*., 2012). For future experimental set-ups, this suggests that small species are especially suitable study organisms because the environmental factor (e.g. temperature or pH) has to be replicated, resulting in a large number of experimental units.

Our experimental design allowed us to identify the range of processes driven by each species in isolation and in combination, under different environmental conditions. We found simple additive species effects across temperatures (Fig.1), with limited effects of richness. This fits with a small but growing body of empirical evidence from similar experimental systems involving single processes (Perkins *et al*., 2010; Reiss *et al*., 2010, 2011). Our tailored statistical models enabled us to explore a range of species richness effects, including facilitation (e.g. Cardinale *et al*., 2002) and resource partitioning (e.g. Cardinale, 2011). We found no evidence, however, that either of these mechanisms influenced polyculture performance in our study.

Not all species promoted each ecosystem process, rather species were functionally different, which meant our set-up was manipulating species richness across functional groups, not within one functional group (cf. Cardinale *et al*. 2006; see Table S2). Nonetheless, some processes, such as leaf decomposition, were driven by all four species and considering this process on its own, species were functionally redundant. Therefore, our study highlights how assemblages can display high within-process redundancy, yet still show high levels of across-process complementarity (cf. Gamfeldt *et al*., 2008). In our experiments, additive effects of functionally different species promoted multifunctionality. That is, variation among species in their contributions to different processes, rather than interactions among species that enhance multiple processes, resulted in positive B–MF relationships.

The high performance of model ‘Type × Temperature’ in explaining single processes highlights that temperature had a strong effect on species performance. Changes in species contributions to different ecosystem processes under environmental change are to be expected given that species have different optima (Vannote & Sweeney, 1980). Indeed, studies in terrestrial systems showed an increase in the number of plant species driving single ecosystem processes under different scenarios of environmental change (Isbell *et al*., 2011); however, these studies did not include temperature. We found that different species and species assemblages promoted multifunctionality at different temperatures. For example, the two best-performing monocultures were *G. pulex* and *S. personatum*, however, which species contributed most to multifunctionality changed across the temperature gradient (Table3). Our study therefore highlights how a larger ‘regional’ species pool is required to maintain ecosystem multifunctionality across a range of environmental conditions.

We hypothesized that when all species contribute to a process, species performance should be related to species body mass and, therefore, functioning should increase with total assemblages biomass (Perkins *et al*., 2010; Reiss *et al*., 2011). Indeed, leaf decomposition and FPOM production were positively correlated with assemblage biomass. However, clear identity effects, not related to body mass, were evident for herbivory and algal production, which were maximized in monoculture by *G. pulex,* the smallest species in the study (Table S2). Variation in the importance of functional traits across different processes meant that no single species or group of species could sustain full multifunctionality (Table3), which therefore increased with species richness under certain contexts.

Correlations between different ecosystem processes were evident in our study (Table S3), consistent with previous B–MF work (Gamfeldt *et al*., 2008, 2013; Zavaleta *et al*., 2010). For example, leaf decomposition, FPOM production and ammonification were all positively correlated, because each of these is a part in a chain of processes typical for decomposition in freshwater systems (Wetzel, 2001). We observed a net uptake of NH_4_^+^ in the microbe-only controls across temperatures but, interestingly, at 15 °C, there was a net release of NH_4_^+^ for invertebrate consumer treatments (Figure S1). This was most likely driven by the different temperature sensitivities of algal and detrital processes. Algal production was largely insensitive to temperature (‘Type’ model outperformed model ‘Type × Temperature’ for this process; Table2) consistent with the notion that substrate supply can override temperature effects (Raven & Geider, 1988). Consequently, the capacity for nitrification (performed by autotrophs) to keep pace with ammonification was exceeded at 15 °C, where rates of leaf decomposition and FPOM production were highest (Figure S4). These results suggest that rising environmental temperatures could alter the balance between different ecosystem processes mediated through detritivore consumers; further work is required, however, to test the generality of these results.

The range of biodiversity levels (up to four species) and number of processes (five) in our experiment meant that the maximum possible slope of the relationship between species richness and the number of processes, greater than a given threshold (*MF*_*t*_), was 1.25 (i.e. 5/4). Where it had its strongest effect (at 5 °C), diversity accounted for 40% of the maximum possible effect on multifunctionality within our experiment, lower than that reported from the terrestrial BIODEPTH studies [range 50–58% (Byrnes *et al*., 2014)]. In our experiment, diversity could not simultaneously drive all processes to their maxima at all three temperatures: the shallower slope at higher thresholds (above 40% at both 5 and 15 °C) indicated that high species richness did not guarantee that all processes were sustained at their highest levels (Fig.2). This observation is consistent with results from terrestrial studies (Byrnes *et al*., 2014), including, e.g. Zavaleta *et al*. (2010) who found that no more than four of seven processes could be simultaneously provided at a threshold of 50%, regardless of the number of species. Taken together with our results, this suggests that: (i) biodiversity tends to promote multifunctionality until trade-offs between different processes mean it is no longer possible to sustain all processes at high levels; and (ii) this phenomenon occurs across different ecosystem types.

This study, which considers ecosystem multifunctionality and environmental contexts simultaneously for the first time, has limitations that should be addressed by future research. For instance, ecosystem responses to changes in temperature will be contingent on the full array of species present within a given system, but the number of species used in this study was relatively low, compared to natural systems (e.g. Woodward *et al*., 2008). The closed nature of the experiment also meant that species could not move in or out of the experimental arenas to track favourable environmental conditions. Furthermore, we assessed the ‘acute’ effects of different thermal regimes on species assemblages, while over longer time scales natural communities would likely change in response to gradually altered environmental conditions, e.g. in favour of warm-tolerant species (Woodward *et al*., 2010). Temperature effects on ecosystem multifunctionality in our study might therefore be overestimates, and worthy of future exploration across a wider range of spatial-temporal scales.

The consequences for ecosystem functioning of biodiversity loss and environmental change are poorly understood, but through manipulating diversity and environmental temperature simultaneously in our experiment, we were able to link the contribution of different assemblages and temperature regimes to a range of single process rates and multifunctionality. The former were reasonably well predicted from monocultures, but because of differences in thermal responses, these were improved still further when information on species performance at different temperatures was included. Although species richness often had negligible effects on single processes, it was far more important when multiple processes and different environmental conditions were considered together: i.e. overall functioning is more contingent on both biodiversity and environmental context than would be inferred from previous generations of B–EF experiments. Consequently, high levels of biodiversity are likely required to sustain multiple ecosystem processes in the face of environmental change anticipated over the next decades.

## Supporting Information

**Data S1.**Length–mass equations for consumer species and analysis of control data. **Figure S1.** Temporal measurement of ammonium (NH_4_^+^) stocks. **Figure S2.** Hierarchy of statistical models tested in the analysis of single ecosystem processes. **Figure S3.** The effects of species richness on ecosystem processes. **Figure S4.** The effects of environmental temperature on ecosystem processes. **Figure S5.** The effect of assemblage biomass on rates of temperature standardized processes. **Table S1.** AIC_c_ and *r*^*2*^ values for statistical models tested in the analysis of single ecosystem processes. **Table S2.** Fitted parameters for model ‘Type × Temperature’. **Table S3.** Correlation matrix for pair wise combinations of ecosystem processes.
