# Supplementary material for: Higher biodiversity is required to sustain multiple ecosystem processes across temperature regimes
Source: Glob Chang Biol. 2014 Aug 18;21(1):396–406. doi: 10.1111/gcb.12688 (PMC4310294; doi:10.1111/gcb.12688)
Supplement: Supplementary file 1 — Data S1.Length–mass equations for consumer species and analysis of control data. Figure S1. Temporal measurement of ammonium (NH4+) stocks. Figure S2. Hierarchy of statistical models tested in the analysis of single ecosystem processes. Figure S3. The effects of species richness on ecosystem processes. Figure S4. The effects of environmental temperature on ecosystem processes. Figure S5. The effect of assemblage biomass on rates of temperature standardized processes. Table S1. AICc and r2 values for statistical models tested in the analysis of single ecosystem processes. Table S2. Fitted parameters for model ‘Type × Temperature’. Table S3. Correlation matrix for pair wise combinations of ecosystem processes. [file gcb0021-0396-sd1.docx]

**Supporting Information**

Higher biodiversity is required to sustain multiple ecosystem processes across temperature regimes

**Daniel M. Perkins, R. A. Bailey, Matteo Dossena, Lars Gamfeldt, Julia Reiss, Mark Trimmer & Guy Woodward**

**S1: Length-mass equations for consumer species and analyses of control data**

Consumer populations were collected from streams in southern England two weeks prior to the start of the experiment and maintained in aerated aquaria at 10°C with leaf litter and benthic biofilms (collected from sampling sites) as food resources.

To determine the biomass of consumer assemblages, high-resolution digital photographs were taken at x100 magnification of the 12 individuals added to each aquarium at the beginning of the experiment. Subsequently, the length of all individuals was measured with the image analysis software Image-Pro® Plus (Media Cybernetics, Inc.) and then converted into dry body mass (mg) using log-log body length-body mass relationships empirically derived for 50 individuals of each species prior to the experiment: *Asellus aquaticus* (*y* = 2.652*x* -1.841, *r*^2^ = 0.94), *Bithynia tentaculata* (*y* = 2.606*x* -4.670, *r*^2^ = 0.68), *Gammarus pulex* (*y* = 3.015*x* -2.242, *r*^2^ = 0.90) and *Sericostoma personatum* (*y* = 1.822*x* -1.029, *r*^2^ = 0.78), where *y* is the body mass in mg and *x* is the length in mm.

Consumer survival was high (93.1 ± 2.5 %) in the experiment. We performed ANOVA to test if process rates in invertebrate treatments differed from microbe-only controls. In addition to the factors Composition and Temperature (Table 1), we fitted Assemblage that had two levels: microbe-only or invertebrates present. For the fixed effects, Composition was nested within Assemblage and an interaction term fitted with Temperature. Block was nested within Lot as random effects (see Materials and Methods). Process rates were significantly greater in invertebrate versus microbe-only treatments in all cases: leaf decomposition (*F_1,45_* = 42.6, *P* < 0.001), herbivory (*F_1,45_* = 57.2, *P* < 0.001), FPOM production (*F_1,45_* = 33.6, *P* < 0.001), algal production (*F_1,45_* = 21.4, *P* < 0.001) and ammonification (*F_1,45_* = 6.1, *P* = 0.017). Leaf mass loss caused by leaching and microbial activity in the first week of the experiment (i.e. prior to the addition of invertebrate consumers) were accounted for by subtracting mean leaf mass loss in this period derived from an additional set of aquaria (*n* = 10 per temperature).

**Figure S1: Temporal measurement of ammonium (NH_4_^+^) stocks.** Net ammonification was calculated from the change in stocks between day *T_0_* (when invertebrate assemblages were introduced) and *T_8_* when stocks peaked. For aquaria containing monocultures of *S. personatum*, stocks peaked at *T_16_* but at a rate indistinguishable to that exhibited between day *T_0_* and *T_8_*. Blue, red and green data points and lines correspond to 15 °C, 10 °C and 5 °C temperature treatments, respectively. Species abbreviations: A, *A. aquaticus*; B, *B. tentaculata*; G, *G. pulex* and S, *S. personatum.*

**Figure S2: Hierarchy of statistical models tested in the analysis of single ecosystem processes,** The diagram is read from the bottom, starting with the smallest model (model ‘Constant’), and going up to the largest model (model ‘Composition × Temperature’). The number of parameters for each model is given in parentheses and models in bold boxes are those listed in Table 1. Each connecting edge on the diagram of models corresponds to a row in the ANOVA table (Table 2). The label (one of a-h) on each edge shows the corresponding effect in Table 1; the number of degrees of freedom for this effect is equal to the difference between the numbers of parameters for the models at the two ends of the edge. We also considered the model ‘Richness × Type’ which has 13 parameters. However, for each of the five single ecosystem processes, a preliminary data analysis showed that the sum of squares for this model was almost as big as that for fitting ‘Composition’, which has 15 parameters, so we decided to exclude the model ‘Richness × Type’.

**Table S1: AIC_c_  and *r^2^* values for statistical models tested in the analysis of single ecosystem processes.**

| **a)** |  | Leaf decomposition | | Herbivory | | FPOM production | | Algal production | | Ammonification | |
| --- | --- | --- | --- | --- | --- | --- | --- | --- | --- | --- | --- |
| **Model** | ***k*** | **AIC_c_** | **Rank** | **AIC_c_** | **Rank** | **AIC_c_** | **Rank** | **AIC_c_** | **Rank** | **AIC_c_** | **Rank** |
| Richness | 4 | 372 | 17 | -581 | 16 | 198 | 18 | -1048 | 15 | 115 | 16 |
| Type | 4 | 359 | 15 | -609 | 6 | 130 | 12 | -1097 | 1 | 106 | 14 |
| Composition | 15 | 387 | 18 | -600 | 11 | 149 | 14 | -1073 | 9 | 125 | 17 |
| Richness+Type | 7 | 366 | 16 | -611 | 5 | 133 | 13 | -1091 | 6 | 113 | 15 |
| Temp. | 3 | 251 | 11 | -583 | 14 | 168 | 15 | -1050 | 14 | 74 | 6 |
| Richness+Temp. | 6 | 258 | 12 | -582 | 15 | 172 | 16 | -1044 | 16 | 80 | 8 |
| Type+Temp. | 6 | 196 | 2 | -613 | 4 | 49 | 7 | -1094 | 4 | 66 | 2 |
| Richness+Type+Temp. | 9 | 202 | 4 | -615 | 3 | 45 | 5 | -1087 | 8 | 73 | 4 |
| Richness×Temp. | 12 | 269 | 13 | -571 | 17 | 185 | 17 | -1041 | 17 | 94 | 11 |
| Type×Temp. | 12 | 191 | 1 | -616 | 2 | 39 | 3 | -1095 | 3 | 60 | 1 |
| Richness×Temp.+Type | 15 | 210 | 6 | -605 | 9 | 55 | 9 | -1093 | 5 | 88 | 10 |
| Type×Temp.+Richness | 15 | 198 | 3 | -619 | 1 | 32 | 1 | -1088 | 7 | 68 | 3 |
| Composition+Temp. | 17 | 223 | 7 | -604 | 10 | 51 | 8 | -1069 | 12 | 79 | 7 |
| Richness×Temp.+Type×Temp. | 21 | 207 | 5 | -607 | 7 | 42 | 4 | -1096 | 2 | 85 | 9 |
| Composition+Type×Temp. | 23 | 222 | 8 | -607 | 8 | 35 | 2 | -1066 | 13 | 74 | 5 |
| Composition+Richness×Temp. | 23 | 235 | 9 | -590 | 13 | 64 | 10 | -1071 | 10 | 98 | 13 |
| Composition+Richness×Temp.+Type×Temp. | 29 | 236 | 10 | -591 | 12 | 48 | 6 | -1071 | 11 | 96 | 12 |
| Composition×Temp. | 45 | 277 | 14 | -532 | 18 | 106 | 11 | -1018 | 18 | 132 | 18 |

| **b)** |  | Leaf decomposition | Herbivory | FPOM production | Algal production | Ammonification |
| --- | --- | --- | --- | --- | --- | --- |
| **Model** | ***k*** | ***r^2^*** | ***r^2^*** | ***r^2^*** | ***r^2^*** | ***r^2^*** |
| Richness | 4 | 0.00 | 0.07 | 0.03 | 0.03 | 0.00 |
| Type | 4 | 0.14 | 0.33 | 0.54 | 0.44 | 0.10 |
| Composition | 15 | 0.14 | 0.45 | 0.59 | 0.47 | 0.19 |
| Richness+Type | 7 | 0.14 | 0.39 | 0.56 | 0.44 | 0.10 |
| Temp. | 3 | 0.73 | 0.07 | 0.29 | 0.04 | 0.35 |
| Richness+Temp. | 6 | 0.73 | 0.13 | 0.31 | 0.04 | 0.36 |
| Type+Temp. | 6 | 0.87 | 0.38 | 0.82 | 0.45 | 0.45 |
| Richness+Type+Temp. | 9 | 0.87 | 0.45 | 0.84 | 0.45 | 0.45 |
| Richness×Temp. | 12 | 0.75 | 0.17 | 0.32 | 0.16 | 0.36 |
| Type×Temp. | 12 | 0.89 | 0.49 | 0.87 | 0.54 | 0.56 |
| Richness×Temp.+Type | 15 | 0.88 | 0.48 | 0.85 | 0.57 | 0.46 |
| Type×Temp.+Richness | 15 | 0.89 | 0.56 | 0.89 | 0.55 | 0.57 |
| Composition+Temp. | 17 | 0.87 | 0.51 | 0.87 | 0.47 | 0.54 |
| Richness×Temp.+Type×Temp. | 21 | 0.91 | 0.59 | 0.90 | 0.66 | 0.57 |
| Composition+Type×Temp. | 23 | 0.90 | 0.62 | 0.91 | 0.57 | 0.66 |
| Composition+Richness×Temp. | 23 | 0.88 | 0.54 | 0.88 | 0.59 | 0.55 |
| Composition+Richness×Temp.+Type×Temp. | 29 | 0.91 | 0.65 | 0.92 | 0.69 | 0.66 |
| Composition×Temp. | 45 | 0.95 | 0.77 | 0.95 | 0.81 | 0.83 |

In a) models are ranked according to AIC_c_ values for each process which measures the goodness of fit for a given model; a low value indicates a good fit with few parameters. AIC_c_ should be used instead of AIC when n/k is less than 40 for the model with the largest value of k (as the case for the ‘Composition × Temperature’ model in our analysis), where *n* is the sample size and *k* is the number of parameters in the model, because it decreases the probability over fitting. Strictly speaking, these information criteria should not be used to compare models where more than one type of random error occurs with the relevant terms in the ANOVA table. Therefore, we split the models into two groups: those without Temperature (upper portion of the table) and with Temperature (lower portion of the table). The ranking on AIC_c_ is correct within each group, but may not be reliable between groups. All models include variation explained by the error term: Blocks[Rooms] (see Table 2 in main text).

**Table S2: Fitted parameters for model ‘Type × Temperature’.**

|  |  |  |  | | | | | |
| --- | --- | --- | --- | --- | --- | --- | --- | --- |
|  |  | Type | Parameter | Leaf decomposition | Herbivory | FPOM production | Algal production | Ammonification |
|  | 5 | A | *b_1_* | 28.29 | 0.0102 | 1.57 | 0.0043 | -0.7799 |
|  | 5 | B | *b_2_* | 27.58 | 0.0417 | 0.86 | 0.0041 | -0.8105 |
|  | 5 | G | *b_3_* | 29.93 | 0.1196 | 0.68 | 0.0089 | -0.9594 |
|  | 5 | S | *b_4_* | 37.65 | 0.0335 | 7.16 | 0.0040 | -0.3943 |
| Temperature (°C) | 10 | A | *c_1_* | 33.08 | 0.0642 | 1.54 | 0.0039 | -1.3582 |
|  | 10 | B | *c_2_* | 33.48 | 0.0546 | 1.73 | 0.0021 | -1.0800 |
|  | 10 | G | *c_3_* | 31.02 | 0.1265 | 1.11 | 0.0138 | -1.3068 |
|  | 10 | S | *c_4_* | 38.34 | 0.0454 | 7.47 | 0.0036 | -0.7497 |
|  | 15 | A | *d_1_* | 39.76 | 0.0530 | 3.20 | 0.0055 | -0.1901 |
|  | 15 | B | *d_2_* | 45.30 | 0.1167 | 4.61 | 0.0036 | -0.3104 |
|  | 15 | G | *d_3_* | 42.01 | 0.1034 | 2.59 | 0.0081 | 0.7948 |
|  | 15 | S | *d_4_* | 56.33 | 0.0078 | 13.69 | 0.0038 | 5.0216 |

Type abbreviations: A, *A. aquaticus*; B, *B. tentaculata*; G, *G. pulex* and S, *S. personatum.* Mean (SE) dry body mass (mg) of consumer species were; 3.80 (0.10), 5.13 (0.14), 3.11 (0.15) and 7.69 (0.18) for *A. aquaticus*, *B. tentaculata*, *G. pulex* and *S. personatum,* respectively*.*

**Figure S3.** **The effects of species richness on ecosystem processes (a-e).** Data points depict mean process rates pooled over temperatures (± 1 SE).

**Figure S4.** **The effects of environmental temperature on ecosystem processes** **(a-e).** Data points depict mean process rates pooled over assemblages (± 1 SE).

**Figure S5:** **The effect of assemblage biomass on rates of temperature standardised (*T*) processes** **(a-e).** Processes were standardised to a common temperature of 10 °C by first calculating the ratio between mean process rates for adjacent temperatures (i.e. between 5 °C and 10 °C and 10 °C and 15 °C; values plotted in Fig. S7). Process values at 5 °C and 15 °C were then multiplied and divided by these empirical ratio’s, respectively. Regression lines represent a statistically significant relationship determined by ordinary least squares regression (1 on 88 degrees of freedom in each case). Dotted lines at y = 0 denote the boundary between the positive and negative delivery of that process.

**Table S3. Correlation matrix for pairwise combinations of ecosystem processes.**

|  | FPOM production | Algal production | Herbivory | Ammonification |
| --- | --- | --- | --- | --- |
| Leaf decomposition | 0.78*** | -0.14 | 0.03 | 0.67*** |
| FPOM production |  | -0.23* | -0.27* | 0.60*** |
| Algal colonization |  |  | 0.36*** | -0.11 |
| Algal grazing |  |  |  | -0.12 |

Pearson correlation coefficients and significant correlations are shown in the upper portion of the matrix. Asterisk denote statistical significance: *** = p< 0.001; ** = p<0 0.01, * = p<0 0.05.
